# Relationship Between Speech Delay and Smart Media in Children: A Systematic Review

**DOI:** 10.7759/cureus.45396

**Published:** 2023-09-17

**Authors:** Manal M Alamri, Muath A Alrehaili, Wejdan Albariqi, Manal S Alshehri, Kholood B Alotaibi, Afnan M Algethami

**Affiliations:** 1 Pediatric, Maternity Children Hospital (MCH), Tabuk, SAU; 2 Pediatric, Ohud Hospital, Madina, SAU; 3 Pediatric Medicine, AL Yamamah Hospital, Second Health Cluster, Riyadh, SAU; 4 Pediatric, King Faisal Hospital, Al-Hasa, SAU; 5 Pediatrics, King Abdullah Medical Complex Hospital, Jeddah, SAU; 6 Pediatric Medicine, King Fahd Medical City (KFMC), Taif, SAU

**Keywords:** smart media, electronic device, screen time, language development, language delay, speech delay

## Abstract

The increasing prevalence of smart media usage among children has raised concerns about its potential impact on various aspects of child development. One such area of worry is speech delay, as early language acquisition is critical for cognitive, social, and educational development. The purpose of this systematic review was to investigate and synthesize available research data in order to determine the association between speech delay and the usage of smart media in children.

To perform this systematic review, a thorough literature search was conducted using relevant keywords in electronic databases, such as PubMed, Scopus, PsycINFO, Google Scholar, Web of Science, and Embase. We included studies published during the last 10 years investigating the impact of smart media on children's speech delay using various research designs.

The findings showed that extended exposure to electronic media for children was negatively associated with expressive vocabulary and language skills in children, in addition to decreased language scores and speech delays. Educational apps and shared media engagement with parents correlated with stronger language skills. The introduction of smart devices at a later stage of development (24 months of age and older) was associated with positive language development, whereas early introduction was associated with speech delay. However, six-month abstinence from devices led to speech improvement in the affected children.

These findings highlight the need to balance interactive screen time and other forms of interaction to enhance speech development.

## Introduction and background

Incorporating smart media devices into children's daily lives has become increasingly common in the quickly evolving digital age, affecting their experiences, interactions, and developmental trajectories [1,2]. Modern parenting has incorporated smart devices to facilitate the parenting of young children [1]. With the introduction of smart media devices (smartphones, tablets, television, computers, game devices) and other electronic devices, children's access to information, engagement with educational content, and connection with the world around them have been transformed. However, in addition to the benefits of modern technologies, there have been concerns raised about their possible impact on several elements of child development, such as language acquisition and communication skills.

Studies have highlighted an association between excessive screen usage and speech delays in young children [3-5]. However, proving a direct cause-and-effect association is complex due to various influencing factors like parenting methods, socioeconomic status, and the overall communication environment [6]. A prominent theory suggests that excessive screen exposure could replace crucial face-to-face interactions between parents/caregivers and children, negatively impacting language development [7-9]. Thus, the American Academy of Pediatrics (AAP) recommends a limited screen time of less than 1-2 hours per day for children under two years of age. It also recommends interactive and educational content for older children who use smart devices to balance screen engagement with other developmental activities [10]. Some research indicates that controlled use of interactive media (engaging and educational) can foster learning, while passive media (watching videos) consumption might negatively affect a child’s development [11].

Language development is a key stage in early childhood cognitive, social, and emotional growth [12]. The intricate process of acquiring language skills is affected by genetics and interactions. Early positive experiences, like responsive interactions and varied language exposure, build strong language abilities [13]. As a result, any factor that may affect the conventional track of language development should be thoroughly investigated. The parallel development in the availability and use of smart media, particularly among society's youngest members, warrants a thorough assessment of how new technologies may interact with children's language learning processes. Research suggests that there might be a negative relationship between increased smart media consumption in early children and expressive language development, with each additional hour of usage reducing expressive language skills [14,15].

While there is some evidence linking excessive smart media exposure to speech delay in children, the exact nature of this association is complex and not entirely understood. Therefore, this systematic review aimed to comprehensively understand the relationship between speech delay and smart media.

## Review

Methods

Search Strategy

The primary research question for this systematic review was: What is the relationship between the use of smart media and the occurrence of speech delay in children? We comprehensively searched databases such as PubMed, Scopus, PsycINFO, Google Scholar, Web of Science, and Embase for relevant studies to answer this question. The search was performed using keywords such as “speech delay,” “language delay,” “language development,” “smart media,” “digital devices,” “smart media,” “screen time,” “electronic devices,” “television,” “smartphone,” “tablet,” computer,” “child development,” “language skills,” “media usage,” and “children,” “toddler,” and “preschooler.” We combined these keywords using Boolean operators (AND, OR) to create effective search queries.

Studies were included if they met the following criteria: (1) focused on children, (2) investigated the relationship between smart media usage and speech delay among children, (3) employed cross-sectional, cohort, case-control, randomization, experimental, meta-analysis, and qualitative research designs, (4) were published in peer-reviewed journals, and (5) were published in English in the last five years. This is to account for the latest evidence available. Studies were excluded if they were case reports, reviews, opinions, and other non-original research articles.

Study Selection

**Figure 1:**
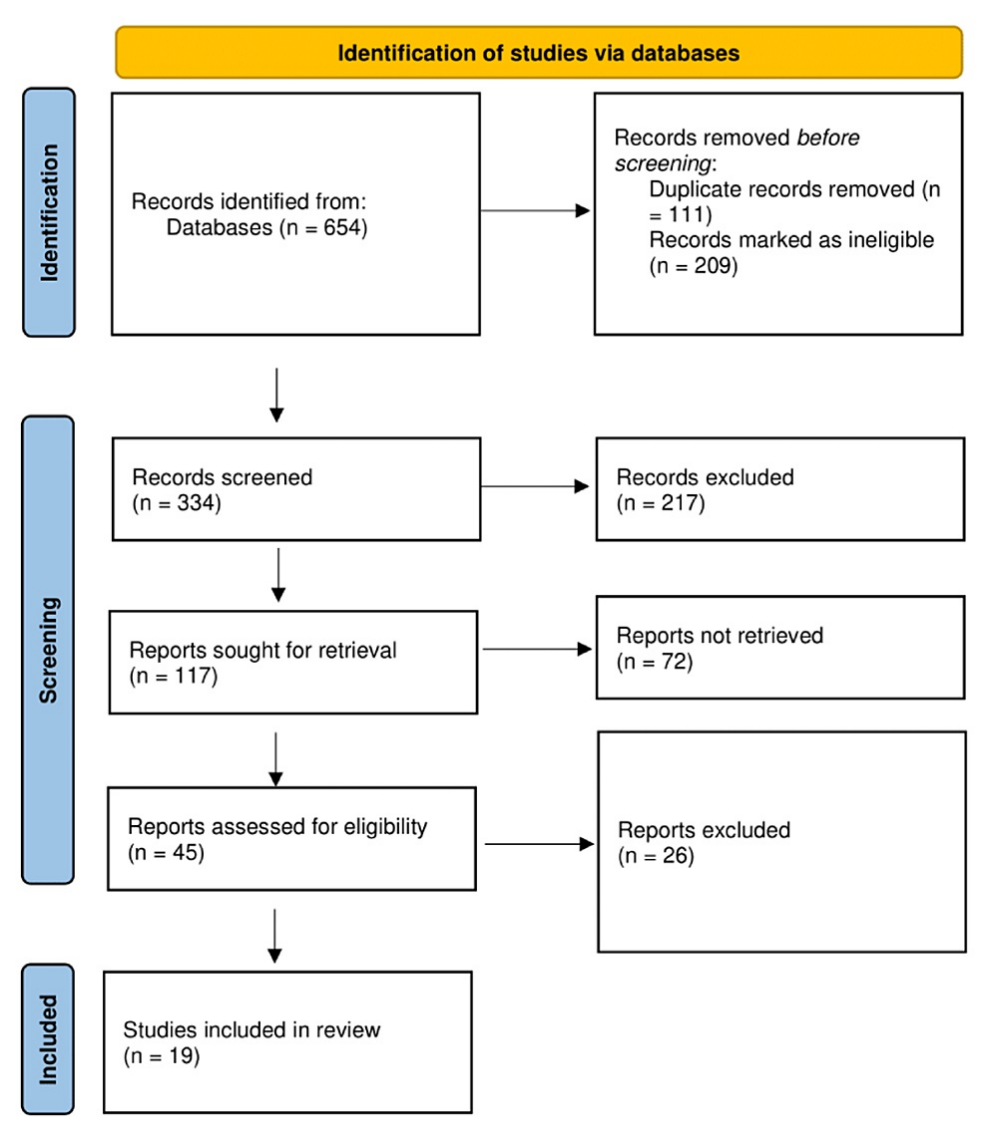
PRISMA flow diagram showing the selection process

Three independent reviewers screened the search results based on titles and abstracts. The full texts of potentially eligible studies were then retrieved and assessed for final inclusion. Any disagreements between the reviewers were resolved through discussion and consultation with a corresponding author if necessary. Figure 1 depicts the process of selecting the included studies. Study selection followed Preferred Reporting Items for Systematic Reviews and Meta-Analyses (PRISMA).

Data Extraction and Quality Assessment

A standardized data extraction form was created to collect relevant information from selected studies, including the first authors’ names, year of publication, study design, and key findings related to the relationship between smart media usage and speech delay. The methodological quality and risk of bias in selected studies were assessed using appropriate tools such as the Newcastle-Ottawa Scale for observational studies [16] and the National Institute of Health (NIH) Study Quality Assessment Tools for other studies [17]. Two reviewers independently performed quality assessments, and any discrepancies were resolved through discussion.

Data Synthesis and Analysis

We synthesized data from selected studies to determine the overall relationship between smart media usage and speech delay among children. Key findings were presented in a table, summarizing the key outcomes reported in each study. A meta-analysis was not feasible due to the potential heterogeneity among the included studies.

Results

The initial search generated 543 results, and after removing duplicates and other articles with exclusion criteria, 334 titles and abstracts were examined. The full-text versions of 117 studies were retrieved from qualifying titles and abstracts. The titles, abstracts, and full texts of 45 papers were deemed eligible and underwent a thorough review, which generated 17 articles fulfilling all inclusion criteria (Table 1). Most of the included articles (11) were cross-sectional studies; two were case-control studies, one was a systematic review and meta-analysis, one used a transversal design, one was a prospective cohort, and the last was a qualitative study.

**Table 1:** Characteristics of the included studies CI: Confidence interval; aOR: Adjusted odd ratio; OR: Odd ratio; TV: Television; SD: Standard deviation

Authors	Year	Title	Study design	Summary of findings
Sola et al. [18]	2022	Tracking Home Language Production and Environment in Children Who Are Deaf or Hard of Hearing	Cross-sectional	For every hour of electronic media exposure, there were reductions in child vocalizations (β = -0.47; 95% CI: -0.71-0.19), conversational turns (β = -0.45; 95% CI: -0.65-0.22), and language development (β = -0.37; 95% CI: -0.61-0.15).
Putu Dianisa [19]	2023	The relationship between screen time and speech delay in 1-2-year-old children	Cross-sectional study	Children who spent over two hours daily using screens had an increased likelihood of experiencing speech delay (aOR=6.15; (95% CI: 2.84-13.30, p < 0.001). Additionally, being male and belonging to a lower socioeconomic background were also associated with speech delay (aOR = 2.67; 95% CI: 1.72-5.60; p = 0.009 and OR=5.49; 95%CI: 2.04-13.93, p = 0.001, respectively).
Ramelan et al. [20]	2019	The Effect of Gadget Use Intensity towards the Speaking Ability of Early Childhood	Cross-sectional study	60% of parents allowed their children to use gadgets without supervision, while only 40% of parents chose to oversee their children's gadget use. Regarding gadget usage frequency, 70% of children engaged with gadgets daily, while the remaining 30% used gadgets selectively. The results of a simple linear regression analysis indicated a significant correlation between the intensity of gadget usage and the speaking proficiency of young children (p<0.001).
Nugrahaet al. [21]	2019	The effect of gadgets on the speech development of toddlers	Qualitative research design using a phenomenological approach	The findings indicated that electronic gadgets had a negative influence on the development of speech in toddlers compared to other children of the same age without gadgets.
Mustonen et al. [22]	2022	Impact of Home Blood Pressure Data Visualization on Hypertension Medical Decision-Making in Primary Care	Cross-sectional study	As children’s time spent on screen or the mothers' screen time increased, their language skills diminished, even after considering the children's age, maternal education level, and birth order. Additionally, there were cumulative and negative associations with the children's lexical and overall language capabilities.
Al Hosani et al. [23]	2023	Home Monitoring of Blood Pressure: Patients’ Perception and Role of the Pharmacist	Case-control study	Electronic gadgets were used by 90.3% of children with speech and language developmental delays. Children possessing a device had an elevated likelihood of experiencing language development issues (OR = 3.94, 95% CI: 1.97-7.84, p < 0.001). In comparison to early initiation (at 12-24 months of age), the introduction of electronic devices at a later stage (25-36 months) exhibited a positive impact on language development (OR = 0.32, 95% CI: 0.13-0.82, p = 0.017). Moreover, children who spend 3 to 4 hours watching television daily were at an increased risk of language delays (OR = 3.21, 95% CI = 1.66-6.17, p < 0.001).
Varadarajan et al. [24]	2021	Prevalence of excessive screen time and its association with developmental delay in children aged <5 years: A population-based cross-sectional study in India	Population-based cross-sectional study	Increased screen time was associated with developmental delays, particularly in language acquisition and communication. In children aged two years and older, a language delay was significantly positively associated with screen time (aOR = 52.92, 95% CI: 12.33-227.21, p < 0.001). For children below two years of age, a language delay was also associated with screen time (aOR = 20.93, 95% CI: 2.68-163.32, p < 0.01).
van den Heuvel et al. [25]	2020	Mobile Media Device Use is Associated with Expressive Language Delay in 18-Month-Old Children	Cross-sectional study	The study analyzed 893 children (mean age 18.7 months, 54.1% male), with most parents reporting no mobile media device usage (77.6%). Among those with reported usage (22.4%), the median daily use was 15.7 minutes. Expressive speech delay prevalence was 6.6%, while the delay of other communication skills was among 8.8% of children. For device users, each additional 30 minutes daily was linked to increased odds of expressive speech delay (aOR = 2.33, 95% CI: 1.25-4.82).
Dy et al. [26]	2023	Measuring effects of screen time on the development of children in the Philippines: A cross-sectional study	Cross-sectional study	Screen time exceeding two hours was associated with a decrease in receptive and expressive language scores. A high likelihood of excessive screen time was observed in children whose parents watched television for over two hours (OR = 4.19, 95% CI: 2.46-7.13, p < 0.001), spent more than two hours browsing the web (OR = 1.75, 95% CI: 1.07-2.88, p = 0.027), and engaged in social media for over two hours (OR = 1.84, 95% CI: 1.15-2.95, p = 0.012). Findings also revealed higher odds of excessive screen time among children who watched TV alone compared to those who watched TV with a parent or adult (OR = 8.56, 95% CI: 1.14-64.07, p = 0.037).
Salunkhe et al. [27]	2021	Influence of Electronic Media on Speech and Language Delay in Children	Cross-sectional study	Speech and language delays were observed in 28.4% of children who spent more than three hours using media.
Acebedo et al. [11]	2020	Impact of the Use of Media Devices within the Family Context on the Language of Preteens	Transversal design	The findings indicated reduced language scores in preteens with increased access to media devices who used them more often and engaged in fewer conversations with their parents. Similarly, preteens who utilized media devices for communication exhibited lower language scores compared to those who used them for educational purposes or learning. These outcomes remained consistent across different socioeconomic backgrounds, sexes, ages, and family languages.
Vrinda et al. [28]	2021	Impact of Screen Time on Communication in Toddlers: A Parental Awareness Survey	Cross-sectional study	The majority (88.5%) of parents acknowledged being conscious of the consequences of increased screen time for children. Visual issues ranked as the primary concern among the outlined effects, while speech delay was the least reported problem. Of all parents, 84% believed that excessive screen time was linked to attention problems, and 93.8% felt that screen time should be limited. However, 56% were unaware of any established guidelines in this regard.
Aziz et al. [29]	2023	Impact of screen exposure on language development among toddlers and preschoolers in Nineveh province	Cross-sectional study	Among the observed cases, 94.9% experienced delayed speech development, while the remaining experienced complete speech loss. A significant correlation was observed between speech delay and screen usage in both groups, particularly those who initiated screen exposure before 24 months of age (P= 0.02) and spent at least four hours daily engaging with screens (P=0.01). Those who spent more hours per day on screens had considerably lower concentrations (p=0.01). After abstaining from these devices for six months, an improvement in speech was noted in 36.7% of cases.
Sundqvist [30]	2021	Growing Up in a Digital World - Digital Media and the Association With the Child’s Language Development at Two Years of Age	Cross-sectional study	The child's language skills were positively correlated with the practice of engaging in reciprocal interactions. The vocabulary and grammar skills showed negative associations with the use of devices by parents during their daily interactions with the child, as well as the extent to which the child watched TV. Additionally, the child's development of practical communication skills had a positive association with parents incorporating devices into child routines, and it was also positively correlated with parents engaging in media activities together with children. In this case, girls had a stronger performance in pragmatic development.
Asikainen et al. [31]	2021	Exposure to electronic media was negatively associated with speech and language development at 18 and 24 months	Prospective cohort	Long periods of exposure to electronic media for both children and parents were associated with a decrease in the child's expressive vocabulary. Our findings revealed that extended screen time (OR = 1.69) and prolonged TV usage (OR = 1.73) were associated with a reduced vocabulary in 18-month-old children. Similarly, increased parental screen time (OR = 2.16) and less frequent shared reading sessions (OR = 0.65) were both associated with a smaller vocabulary in 24-month-old children.
Venker et al. [32]	2022	Electronic Toys Decrease the Quantity and Lexical Diversity of Spoken Language Produced by Children With Autism Spectrum Disorder and Age-Matched Children With Typical Development	Case-control study	For both children with autism spectrum disorder (ASD) (p = 0.025) and typically developing (TD) children (p = 0.004), the average count of child utterances per minute was significantly reduced during electronic toy play compared to traditional toy play. Similarly, the mean number of distinct words per minute was significantly lower during electronic toy play in both children with ASD (p = 0.021) and children with TD (p = 0.005).
Madigan et al. [15]	2020	Associations Between Screen Use and Child Language Skills: A Systematic Review and Meta-analysis	Systematic Review and Meta-analysis	Increased screen usage duration (hours per session) was associated with reduced language proficiency (r = −0.14; 95% CI: −0.18−0.10). On the other hand, enhanced screen quality, like educational programs (r = 0.13; 95%CI: 0.02-0.24, and joint usage with parents (r = 0.16; 95%CI: 0.07-0.24), was associated with stronger child language abilities. Beginning screen usage at a later age was correlated with improved child language skills (r = 0.17; 95%CI: 0.07-0.27) compared to early-age usage.

One study found that electronic gadgets were used by 90.3% of children with speech and language developmental delays [23]. This high prevalence of use of electronic gadgets in children with language delays was also reported in other included studies. Most users (70%) engage with gadgets daily, while the remaining 30% use gadgets selectively [20]. One study reported a median daily use of 15.7 minutes among 22.4% of electronic device users, while 77.6% were not users [25].

The findings from the included studies indicated that media devices affect the development of speech in children [11,15,21,22,27,29-32]. There was a high likelihood of language development problems in children possessing electronic devices (Odd ratio [OR] = 3.94, 95% confidence interval [CI]: 1.97-7.84, p < 0.001) [23]. It was found that long periods of exposure to electronic media for both children and parents were associated with a decrease in the child's expressive vocabulary and poor language skills, including lexical skills [22,31], indicating that parental screen time also affects a child’s language abilities. In this regard, parental use of electronic devices influences similar behaviors in children. One study found a high likelihood of excessive screen time in children whose parents watched television for over two hours (OR = 4.19, 95% CI: 2.46-7.13, p < 0.001), spent more than two hours browsing the web (OR = 1.75, 95% CI: 1.07-2.88, p = 0.027), and engaged in social media for over two hours (OR = 1.84, 95% CI: 1.15-2.95, p = 0.012) [26].

One study found that vocabulary and grammar skills were negatively associated with parents' use of electronic devices when interacting with the child, as well as watching TV and less frequent shared reading sessions [30,31]. Another study found that increased media device access led to lower language scores in preteens who used them frequently, especially if conversations with parents were limited by the device use [11]. For children with autism spectrum disorders and children without autism, the average count of child utterances per minute and the mean number of distinct words per minute were significantly reduced during electronic toy play compared to traditional toy play [32]. Language acquisition delay was also found to be associated with increased screen time in children aged more than two years (Adjusted OR [aOR] = 20.93, 95% CI: 2.68-163.32, p < 0.01) and less than two years (aOR = 52.92, 95% CI: 12.33-227.21, p < 0.001, respectively) [24]. For every hour of electronic media exposure, there were reductions in child vocalizations, conversational turns, and language development [18]. On the other hand, one study found that each additional 30 minutes of daily screen time was linked to increased odds of expressive speech delay (aOR = 2.33, 95% CI: 1.25-4.82) [25], confirming that increased screen usage duration (hours per session) was associated with reduced language proficiency (r = −0.14; 95% CI: −0.18-[−0.10]) reported in another study [15].

Studies established a correlation between the intensity of gadget usage and the speaking proficiency of young children, with a higher risk of language delay in children with more gadget use intensity [15,19,20,23,25,26,29]. Screen time exceeding two hours and watching TV for 3 to 4 hours daily were associated with a decrease in receptive and expressive language scores and speech delays [19,23,26]. Moreover, findings also revealed higher odds of screen time among children who watched alone compared to those who viewed with a parent or adult (OR = 8.56, 95% CI: 1.14-64.07, p = 0.037) [26], indicating the parental influence on screen time among children. This influence can also be positive since the child's development of practical communication skills was found to have a positive association with parents incorporating devices into child routines and engaging in media activities together with children [30].

Three studies reported the influence of sex in the association between smart media and speech delay [11,19,30]. While one study reported that male sex and lower socioeconomic background were associated with speech delay (aOR = 2.67; 95%CI: 1.72-5.60; p = 0.009 and OR = 5.49; 95%CI: 2.04-13.93, p = 0.001, respectively) [19], another study found no influence of socioeconomic backgrounds, sex, age, and family languages [11]. One study found that girls had stronger performance in pragmatic development when parents incorporated electronic devices into children’s routines, helping them to develop communication skills [30]. This positive impact of smart media devices when used appropriately is also confirmed by another study that showed that using them for educational programs (r = 0.13; 95%CI: 0.02-0.24, and joint usage with parents (r = 0.16; 95%CI: 0.07-0.24) were associated with stronger child language skills [15]. This is also consistent with another study showing that using electronic devices for communication correlated with lower language scores compared to using them for educational purposes [11].

One study found that screen exposure at an early stage of life, before two years of age, was significantly associated with speech delay (p = 0.02) [29]. On the other hand, introducing electronic devices at a later stage of development was found to positively affect a child’s language development [15], with introducing them at 25-36 months of age being positively associated with language skills (p = 0.017) [23]. Though smart media devices were associated with speech delay and other language problems among children, only one study reported that abstaining from these devices for six months was associated with speech improvement in 36.7% of children with speech delay [29].

One study evaluating parental awareness found that most parents (88.5%) were aware of the consequences of increased screen time. However, speech delay was the least common problem they were concerned about, while vision problems were their most common concern [28]. Other problems reported by parents were related to attention, consistent with another study that found a significant association between screen time and low concentration among children (p = 0.01) [29].

Dicussion

This systematic review explored the association between smart media use and speech delay in children. By synthesizing existing information, this systematic review contributes to the present knowledge base, inspires future research projects, and provides insights for parents, educators, and policymakers concerned about the impact of smart media on child development.

The findings showed that most children used smart media devices or watched TV. Similarly, another previous study found that almost a third (31.1%) of the children with an average daily usage time of 3.1 hours experienced a speech delay, though the association between smart device usage duration and speech delay was insignificant (p = 0.538) [33]. Other studies found that children who use smart media devices for extended periods of time are more likely to have speech delays than children who do not use these devices as much. For example, a study from Korea reported that there was a proportional increase in the risk of language delay with each increment in toddlers' TV-watching time (p = 0.004) [12], aligning with our findings, and another previous study also showed that the extent of electronic media exposure was negatively associated with receptive language skills [34]. This is consistent with our findings that screen time and watching TV for 3 to 4 hours daily were associated with decreased receptive and expressive language scores and speech delays.

The association between smart media use and speech delays could be explained in a variety of ways. Children who use smart media devices may be less likely to engage in activities that encourage speech development, such as talking to their parents and siblings [35,36]. This is supported by our findings showing that parental usage of smart devices was also associated with poor vocabulary and lower lexical skills in their children. Our findings also showed that when smart devices interfere with conversations with parents, the children score lower in language acquisition. When children spend a long time on screens, they are not connecting with others, which limits their opportunities to acquire new words and develop communication skills [13]. This leads to poor vocabulary, reductions in child vocalizations, conversational turns, and overall speech delay, especially in children under five, as indicated by our findings. The age of exposure is crucial since we found that children below two years of age who are exposed to smart media devices have higher odds of language delay than those over two years of age (aOR = 53.9 vs. aOR = 20.9), aligning with previous studies [37,38]. We also found that exposure at a later stage of development was less detrimental to language skills, implying that advanced age might be protective. This is confirmed by previous studies that reported age as a protective factor for early language delay [39,40]. There is evidence that parental screen duration can, directly and indirectly, affect children’s screen duration and the parent-child relationship [41]. Similarly, our findings showed a high likelihood of excessive screen time in children whose parents’ screen time was over two hours. However, the involvement of parents in supervising and regulating the time and content of the child was positively associated with language development in children. Co-viewing, where parents interact with and discuss the information with their children, can improve the educational value of screen time [42]. Our systematic review showed that watching with caregivers or parents reduced screen time for children, and parents guiding children to use smart devices for educational purposes and interactive content were associated with improved language and communication skills.

Many smart media apps and games are intended to be passive experiences in which children watch or listen without interacting [43]. This type of content does not give children as many opportunities to improve their speech skills as more participatory games like playing with toys or talking to friends [43,44], which limits their capacity to enrich their language skills. Our systematic review showed that electronic toy play significantly reduces the average count of child utterances and the mean number of distinct words per minute compared to traditional toy play. This might be because electronic toys do not support caregiver-child interactions and creativity compared to traditional toys [45]. Though our findings did not find any influence of socio-demographic factors in the relationship between smart media and speech delay other than sex and age, previous research showed that ethnicity, maternal language, hearing conditions, household size, and income were predictors of speech delay [39]. We found that girls performed stronger in pragmatic language, indicating that the male sex might be more susceptible to language development problems. Previous studies also found that male sex was a factor in speech delay [39,46].

This systematic review has some limitations for consideration. We excluded many articles as most search results had been published more than 10 years ago, were conducted on adults, and were non-original articles (mostly reviews and case reports), all of which constituted exclusion criteria for our study. Additionally, we restricted our inclusion criteria to articles published in English and in peer-reviewed journals, with a clear statement of the study designs used. This led to more articles not fulfilling inclusion criteria and consequently being excluded. Another limitation was the heterogeneity among the included studies, making meta-analysis not feasible. Therefore, extensive longitudinal studies are recommended to further understand the impact of smart media devices on children's language, cognitive, and physical development. 

## Conclusions

This systematic review showed that excessive and unsupervised smart media use may contribute to speech delay. The later the smart device is introduced, the better the language development. Depending on how technology is integrated into a child's daily routine, smart media can help or impede language development. Maintaining a balance between interactive screen time and other forms of engagement, such as face-to-face interactions and outdoor play, frequent conversation, daily reading, and interactive games, is ultimately critical for promoting speech development and counteracting the negative effects of smart media. The findings showed that best practices for co-viewing with parents and caregivers may steer children toward a good, engaging, and high-quality, age-appropriate media experience that complements their language development path.

## References

[1] Chen C, Chen S, Wen P, Snow CE (2020). Are screen devices soothing children or soothing parents? Investigating the relationships among children’s exposure to different types of screen media, parental efficacy and home literacy practices. Comput Hum Behav.

[2] Barr R (2019). Growing up in the digital age: early learning and family media ecology. Curr Dir Psychol Sci.

[3] Muppalla SK, Vuppalapati S, Reddy Pulliahgaru A, Sreenivasulu H (2023). Effects of excessive screen time on child development: an updated review and strategies for management. Cureus.

[4] Veronica N, Gupita N (2020). Electronic media and language development of early childhood. J Phys: Conf Ser.

[5] Karani NF, Sher J, Mophosho M (2022). The influence of screen time on children's language development: A scoping review. S Afr J Commun Disord.

[6] Panjeti-Madan VN, Ranganathan P (2023). Impact of screen time on children’s development: cognitive, language, physical, and social and emotional domains. MTI.

[7] (2017). Screen time and young children: promoting health and development in a digital world. Paediatr Child Health.

[8] Braune-Krickau K, Schneebeli L, Pehlke-Milde J, Gemperle M, Koch R, von Wyl A (2021). Smartphones in the nursery: Parental smartphone use and parental sensitivity and responsiveness within parent-child interaction in early childhood (0-5 years): A scoping review. Infant Ment Health J.

[9] Kildare CA, Middlemiss W (2017). Impact of parents mobile device use on parent-child interaction: A literature review. Comput Hum Behav.

[10] (2013). Children, adolescents, and the media. Pediatrics.

[11] Acebedo L, Buil-Legaz L, Adrover-Roig D, Aguilar-Mediavilla E (2020). Impact of the use of media devices within the family context on the language of preteens. Children (Basel).

[12] Byeon H, Hong S (2015). Relationship between television viewing and language delay in toddlers: evidence from a Korea national cross-sectional survey. PLoS One.

[13] Gómez E, Strasser K (2021). Language and socioemotional development in early childhood: The role of conversational turns. Dev Sci.

[14] Monteiro R, Ferreira S, Fernandes S, Rocha N (2023). Does digital media use contribute to decreased expressive language skills of pre-school-aged children? An exploratory study in Portuguese children. Somatosens Mot Res.

[15] Madigan S, McArthur BA, Anhorn C, Eirich R, Christakis DA (2020). Associations between screen use and child language skills: a systematic review and meta-analysis. JAMA Pediatr.

[16] Lo CK, Mertz D, Loeb M (2014). Newcastle-Ottawa Scale: comparing reviewers' to authors' assessments. BMC Med Res Methodol.

[17] NIH NIH (2023). NIH: Study quality assessment tools. National Institute of Health.

[18] Sola AM, Brodie KD, Stephans J, Scarpelli C, Chan DK (2022). Tracking home language production and environment in children who are deaf or hard of hearing. Otolaryngol Head Neck Surg.

[19] Dewi P, Soetjiningsih Soetjiningsih, Subanada I, Utama M, Artana W, Arimbawa M, Nesa N (2023). The relationship between screen time and speech delay in 1-2-year-old children. GSC Adv Res Rev.

[20] Ramelan H, Novianti R, Kurnia R The effect of gadget use intensity towards the speaking ability of early childhood. Riau University.

[21] Nugraha A, Izah N, Nurul Hidayah S, Zulfiana E, Qudriani M (2019). The effect of gadget on speech development of toddlers. J Phys: Conf Ser.

[22] Mustonen R, Torppa R, Stolt S (2022). Screen time of preschool-aged children and their mothers, and children's language development. Children (Basel).

[23] Al Hosani SS, Darwish EA, Ayanikalath S, AlMazroei RS, AlMaashari RS, Wedyan AT (2023). Screen time and speech and language delay in children aged 12-48 months in UAE: a case-control study. Middle East Curr Psychiatry.

[24] Varadarajan S, Govindarajan Venguidesvarane A, Ramaswamy KN, Rajamohan M, Krupa M, Winfred Christadoss SB (2021). Prevalence of excessive screen time and its association with developmental delay in children aged <5 years: A population-based cross-sectional study in India. PLoS One.

[25] van den Heuvel M, Ma J, Borkhoff CM (2019). Mobile media device use is associated with expressive language delay in 18-month-old children. J Dev Behav Pediatr.

[26] Dy AB, Dy AB, Santos SK (2023). Measuring effects of screen time on the development of children in the Philippines: a cross-sectional study. BMC Public Health.

[27] Salunkhe S, Bharaswadkar R, Patil M, Agarkhedkar S, Pande V, Mane S (2021). Influence of electronic media on speech and language delay in children. Med J DY Patil Vidyapeeth.

[28] Vrinda R, Reji MM, Sanjeevan SS (2021). Impact of screen time on communication in toddlers: a parental awareness survey. Lang Ind.

[29] Aziz ZW, Aljammas EK, Al-Allaf LIK (2023). Impact of screen exposure on language development among toddlers and preschoolers in Nineveh province. MMSL.

[30] Sundqvist A, Koch FS, Birberg Thornberg U, Barr R, Heimann M (2021). Growing up in a digital world - digital media and the association with the child's language development at two years of age. Front Psychol.

[31] Asikainen M, Kylliäinen A, Mäkelä TE, Saarenpää-Heikkilä O, Paavonen EJ (2021). Exposure to electronic media was negatively associated with speech and language development at 18 and 24 months. Acta Paediatr.

[32] Venker CE, Johnson JR (2022). Electronic toys decrease the quantity and lexical diversity of spoken language produced by children with autism spectrum disorder and age-matched children with typical development. Front Psychol.

[33] Borajy S, Albkhari D, Turkistani H, Altuwairiqi R, Aboalshamat K, Altaib T, Almehman W (2019). Relationship of electronic device usage with obesity and speech delay in children. FMPCR.

[34] Ambrose SE, VanDam M, Moeller MP (2014). Linguistic input, electronic media, and communication outcomes of toddlers with hearing loss. Ear Hear.

[35] Hosokawa R, Katsura T (2018). Association between mobile technology use and child adjustment in early elementary school age. PLoS One.

[36] Guellai B, Somogyi E, Esseily R, Chopin A (2022). Effects of screen exposure on young children's cognitive development: a review. Front Psychol.

[37] Mondal N, Bhat BV, Plakkal N, Thulasingam M, Ajayan P, Poorna DR (2016). Prevalence and risk factors of speech and language delay in children less than three years of age. J Compr Ped.

[38] Tan S, Mangunatmadja I, Wiguna T (2019). Risk factors for delayed speech in children aged 1-2 years. PI.

[39] Cheung RW, Willan K, Dickerson J, Bowyer-Crane C (2023). Risk factors for early language delay in children within a minority ethnic, bilingual, deprived environment (Born in Bradford's Better Start): a UK community birth cohort study. BMJ Paediatr Open.

[40] Pérez-Pereira M (2021). Prevalence of language delay among healthy preterm children, language outcomes and predictive factors. Children (Basel).

[41] Li H, Luo W, He H (2022). Association of parental screen addiction with young children's screen addiction: a chain-mediating model. Int J Environ Res Public Health.

[42] Blumberg FC, Brooks PJ (2017). Cognitive development in digital contexts. Elsevier.

[43] Yang CC (2016). Instagram use, loneliness, and social comparison orientation: interact and browse on social media, but don't compare. Cyberpsychol Behav Soc Netw.

[44] Stevic A, Matthes J (2021). A vicious circle between children’s non-communicative smartphone use and loneliness: Parents cannot do much about it. Telemat Inform.

[45] Hassinger-Das B, Quinones A, DiFlorio C, Schwartz R, Talla Takoukam NC, Salerno M, Zosh JM (2021). Looking deeper into the toy box: understanding caregiver toy selection decisions. Infant Behav Dev.

[46] Collisson BA, Graham SA, Preston JL, Rose MS, McDonald S, Tough S (2016). Risk and protective factors for late talking: an epidemiologic investigation. J Pediatr.

